# IL6-R blocking with tocilizumab in critically ill patients with hemophagocytic syndrome

**DOI:** 10.1186/s13054-020-02878-7

**Published:** 2020-04-22

**Authors:** Etienne Dufranc, Arnaud Del Bello, Julie Belliere, Nassim Kamar, Stanislas Faguer, on behalf of the TAIDI (Toulouse Acquired Immune Deficiency and Infection) study group, Etienne Dufranc, Arnaud Del Bello, Julie Belliere, Nassim Kamar, Stanislas Faguer

**Affiliations:** 1 Département de Néphrologie et Transplantation d’organes, Centre Hospitalier Universitaire de Toulouse, F-31000 Toulouse, France; 2 Institut National de la Santé et de la Recherche Médicale, U1043, IFR-BMT, Hôpital Purpan, F-31000 Toulouse, France; 3 Université Paul Sabatier – Toulouse 3, F-31000 Toulouse, France; 4 Institut National de la Santé et de la Recherche Médicale, U1048, Institut des Maladies Métaboliques et Cardiovasculaires, Hôpital Rangueil, F-31000 Toulouse, France

**Keywords:** IL-6, Tocilizumab, Hemophagocytic lymphohistiocytosis, HLH

To the Editor,

Hemophagocytic lymphohistiocytosis (HLH) is a rare life-threatening hematological disorder characterized by uncontrolled activation of CD8^+^ T cells and NK cells, cytokine storm (including overproduction of interleukine-6 (IL6)), and uncontrolled hemophagocytosis leading to severe organ dysfunction [1]. Several causes of HLH have been identified, including infection, cancer, drugs, and autoimmune diseases [1]. Diagnosis of HLH is challenging, and the *H*-score may help to better identify patients with reactive HLH [2].

A combination of dexamethasone, etoposide, and treatment of the underlying cause is the cornerstone of treatment for severe forms of HLH [1]. Because some patients may develop refractory or relapsing HLH, alternative treatments targeting specific immune pathways or cytokine signaling have been tested [1]. These approaches also aim to avoid long-lasting etoposide-induced neutropenia in patients with bone marrow failure or after transplantation.

Tocilizumab, a monoclonal antibody targeting the receptor of IL6, fully reverses the multi-organ failure and the cytokine profile of the CAR-T cell-induced cytokine-release syndrome [3]. This prompted some groups including ours to treat severe HLH secondary to acute autoimmune disease with tocilizumab [4]. Targeting one of the major cytokines that orchestrate the cytokine storm may be an alternative to etoposide in patients with HLH not related to hematological malignancies.

In the herein study, we reviewed the outcomes of nine critically ill patients who received tocilizumab to treat HLH (Table 1). Eight of them received at least one organ support. Median *H*-score was 208 (probability of HLH according to the score, 92.5%), and all patients had at least 4 to 7 criteria of the modified 2009 HLH criteria (genetic testing and NK cell activity were not available; sCD25 was tested in one patient). Causes of HLH were multiples: autoimmune diseases in four, infection (bacterial or viral) in three, and idiopathic in two. In addition to tocilizumab (8 mg/kg once, intravenously), five patients received concomitant treatment with dexamethasone (*n* = 4), cyclophosphamide (*n* = 2), or intravenous immunoglobulins (*n* = 1). Remission was observed in 8/9 patients after tocilizumab (88.9%) whereas one developed refractory HLH, also unresponsive to rescue therapy with etoposide. Ferritin progressively decreased over the first 2 weeks (Fig. 1). One patient relapsed during the hospitalization and successfully received etoposide, but she ultimately died from unrelated gut ischemia. No patient developed profound neutropenia (< 500 cells/mm^3^), except one who had also received cyclophosphamide. During the hospitalization, four patients died (sepsis-related multi-organ failure *n* = 1; refractory HLH *n* = 1; organ support limitation *n* = 2). None developed HLH relapse beyond the current hospitalization. Cytomegalovirus prophylaxis was pursued at least 3 months in transplant recipients.

**Table 1:** Characteristics and outcomes of nine patients with hemophagocytic syndrome who received tocilizumab. *M*, male; *F*, female; *CAPS*, catastrophic antiphospholipid syndrome; *TMA*, thrombotic microangiopathy; *PVB19*, parvovirus B19; *LGL*, large granular lymphocyte leukemia; *HLH*, hemophagocytic lymphohistiocytosis; *DXM*, dexamethasone; *CYC*, cyclophosphamide; *IVIg*, intravenous immunoglobulins; *AIHA*, autoimmune hemolytic anemia; *SCT*, stem cell transplantation; *MMF*, mycofenolate mofetil; *Cst*, corticosteroids; *CsA*, ciclosporin-A; *CR*, complete response; *IS*, immunosuppressive regimen; *MV*, mechanical ventilation; *RRT*, renal replacement therapy; *VD*, vasopressive drugs; *OSL*, organ support limitations; mHLH2009, modified 2009 HLH criteria

	Age	Gender	Cause of HLH	Underlying immunodeficiency	On-going IS at the onset	***H***-score/mHLH2009	Other HLH therapy	Organ supports	HLH response	Relapse	Outcomes
**1**	59	M	Multiple autoimmune disorders^a^, TMA	Cst	Cst	248 (99.3%)/7	DXM, CYC	MV, RRT, VD	CR	No	Alive
**2**	43	M	Septicemia	Allogenic SCT	Cst	220 (96.3%)/5	No	MV, RRT, VD	CR	No	Death (septic shock; OSL)
**3**	23	F	Idiopathic	Heart transplantation	Tacrolimus, MMF, Cst, IVIg	210 (93%)/5	No	RRT, VD	CR	No	Alive
**4**	60	M	Infections (varicella zoster virus, parvovirus B19, HSV-2), septicemia	Heart transplantation	Tacrolimus, MMF, Cst	188 (78%)/5	Etoposide	MV, RRT, VD	None	–	Death (septic shock, aspergillosis, refractory HLH)
**5**	52	M	Parvovirus B19 and CAPS	No	No	208 (92.5%)/5	IVIg, DXM	MV, RRT, VD	CR	No	Alive
**6**	53	M	Idiopathic	Liver transplantation	Tacrolimus, MMF, Cst	18 (79%)/5	No	MV, RRT	CR	No	Alive
**7**	66	F	Overlap syndrome, TMA	Cst	Cst, rituximab	186 (75.8%)	DXM	MV, RRT, VD	CR	Yes (etoposide)	Death (gut ischemia; OSL)
**8**	57	F	Refractory AIHA	T-LGL, B cell lymphoma	Dxm, CsA,	188 (78%)/4	CYC, DXM	MV, RRT, VD	CR	No	Death (septic shock, refractory AIHA)
**9**	25	F	*S. hominis* bacteriemia, HSV-1	Kidney and liver transplantation	Tacrolimus, MMF, Cst	218 (95.8%)/6	No	_	CR	No	Alive

^a^Patient 1 was described in reference [3]. He was first hospitalized for thrombotic microangiopathy associated with autoimmunity and symptoms of rheumatoid arthritis, anti-synthetase syndrome, systemic lupus erythematosus, cryoglobulinemia, and Sjogren syndrome

**Fig. 1:**
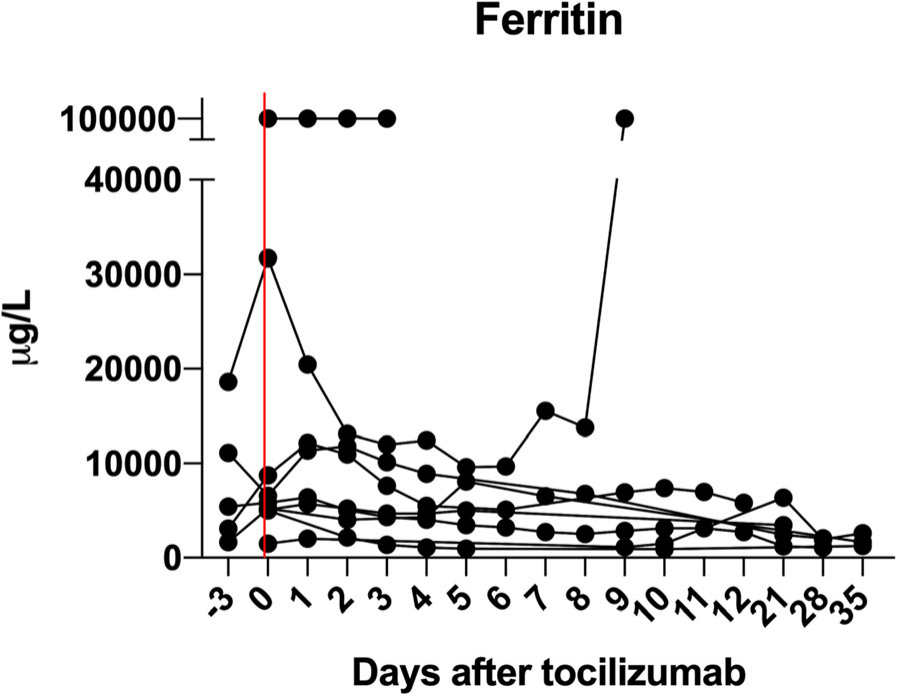
Ferritin concentration after tocilizumab

In critically ill patients with severe forms of HLH, etoposide rapidly reverses cytokine storm and improves clinical condition [1]. HLH 94 and 2004 protocols were developed for children with primary HLH (50% successes), but adult patients may be more at risk to develop chemotherapy toxicities [1]. Alternatives should thus be discussed in adult patients with chemotherapy-induced bone marrow failure, underlying autoimmune diseases requiring cytotoxic agents, or with a moderate form of HLH not related to hematological malignancies. In line with this need, the JAK1/2 inhibitor ruxolitinib was tested in a mouse model of genetic HLH. Its benefits were confirmed in patients with reactive HLH [5], but the oral administration may preclude its pharmacokinetic in critically ill patients requiring mechanical ventilation. Due to its intravenous administration, tocilizumab may thus be a valuable alternative after ruling out on-going bacterial or fungal sepsis.

In conclusion, IL-6-R blockade with tocilizumab may be an alternative in critically ill patients with moderate forms of HLH. Whether such beneficial effects may also be observed in the subset of patients with a cytokine-related syndrome induced by the recently emerging SARS-CoV2 virus remains to be addressed.

## Data Availability

The authors stated that all the data are available upon request.
